# Quantitative assessments of water-use efficiency in Temperate Eurasian Steppe along an aridity gradient

**DOI:** 10.1371/journal.pone.0179875

**Published:** 2017-07-07

**Authors:** Yizhao Chen, Jianlong Li, Weimin Ju, Honghua Ruan, Zhihao Qin, Yiye Huang, Nasreen Jeelani, José Padarian, Pavel Propastin

**Affiliations:** 1 Joint Innovation Center for Modern Forestry Studies, College of Biology and Environment, Nanjing Forestry University, Nanjing, China; 2 School of Life Science, Nanjing University, Nanjing, PR China; 3 International Institute for Earth System Sciences, Nanjing University, Nanjing, China; 4 Institute of Agro-Resources and Regional Planning, Chinese Academy of Agricultural Sciences, Beijing, PR China; 5 School of Atmospheric Sciences, Nanjing University, Nanjing, PR China; 6 Department of Ecology, Nanjing University, Nanjing, PR China; 7 Faculty of Agriculture and Environment, University of Sydney, Sydney, Australia; 8 Institute of Geography, Georg-August University Göttingen, Göttingen, Germany; 9 Department of Bioclimatology, Büsgen-Institute, Georg-August University Göttingen, Göttingen, Germany; Xinjiang Institute of Ecology and Gepgraphy, CHINA

## Abstract

Water-use efficiency (WUE), defined as the ratio of net primary productivity (NPP) to evapotranspiration (ET), is an important indicator to represent the trade-off pattern between vegetation productivity and water consumption. Its dynamics under climate change are important to ecohydrology and ecosystem management, especially in the drylands. In this study, we modified and used a late version of Boreal Ecosystem Productivity Simulator (BEPS), to quantify the WUE in the typical dryland ecosystems, Temperate Eurasian Steppe (TES). The Aridity Index (AI) was used to specify the terrestrial water availability condition. The regional results showed that during the period of 1999–2008, the WUE has a clear decreasing trend in the spatial distribution from arid to humid areas. The highest annual average WUE was in dry and semi-humid sub-region (DSH) with 0.88 gC mm^-1^ and the lowest was in arid sub-region (AR) with 0.22 gC mm^-1^. A two-stage pattern of WUE was found in TES. That is, WUE would enhance with lower aridity stress, but decline under the humid environment. Over 65% of the region exhibited increasing WUE. This enhancement, however, could not indicate that the grasslands were getting better because the NPP even slightly decreased. It was mainly attributed to the reduction of ET over 70% of the region, which is closely related to the rainfall decrease. The results also suggested a similar negative spatial correlation between the WUE and the mean annual precipitation (MAP) at the driest and the most humid ends. This regional pattern reflected the different roles of water in regulating the terrestrial ecosystems under different aridity levels. This study could facilitate the understanding of the interactions between terrestrial carbon and water cycles, and thus contribute to a sustainable management of nature resources in the dryland ecosystems.

## Introduction

According to the latest IPCC report [1], global surface temperature increased by 0.85°C in last 100 years and will continue to rise by 0.3°C to 4.8°C relative to 1986–2005., Water shortage in arid/semi-arid regions is reported to become more serious due to various factors [2]. Furthermore, because of the tight interactions between the terrestrial carbon and hydrological cycles, this issue will also greatly affect the regional and global carbon budgets [3–5]. Water-use efficiency (WUE), the ratio of vegetation production (GPP, NPP or NEP) to evapotranspiration (ET), offers an integrated view to the carbon and water cycles in terrestrial ecosystems and their responses to climatic change [6–8].

Temperate Eurasian Steppe (TES), the largest grassland belt in the world, stretches from Hungary to the northeast of China, lying between the boreal forest in Russia and the vast desert area in Central Asia. It spans around one-fifth of the longitude with an area of over 1.3×10^7^ km^2^ [9]. This eco-zone plays a significant role in the regional and global carbon and hydrological circulations, and also offers essential ecosystem services to maintain life [10–12]. Much of the region is controlled by the continental climate. With large spatiotemporal variations in precipitation, the water shortage issue is severe [13, 14]. Recent climate change draws extra pressure on this region. Observed evidence is that regional annual precipitation decreased during the past 30 years [15]. Such trend increases the regional drought stress due to the higher water loss from the land surface. According to the recent analysis from Mohammat, Wang [16], the possibility of summer drought is enhancing since the 1990s.

**Fig 1:**
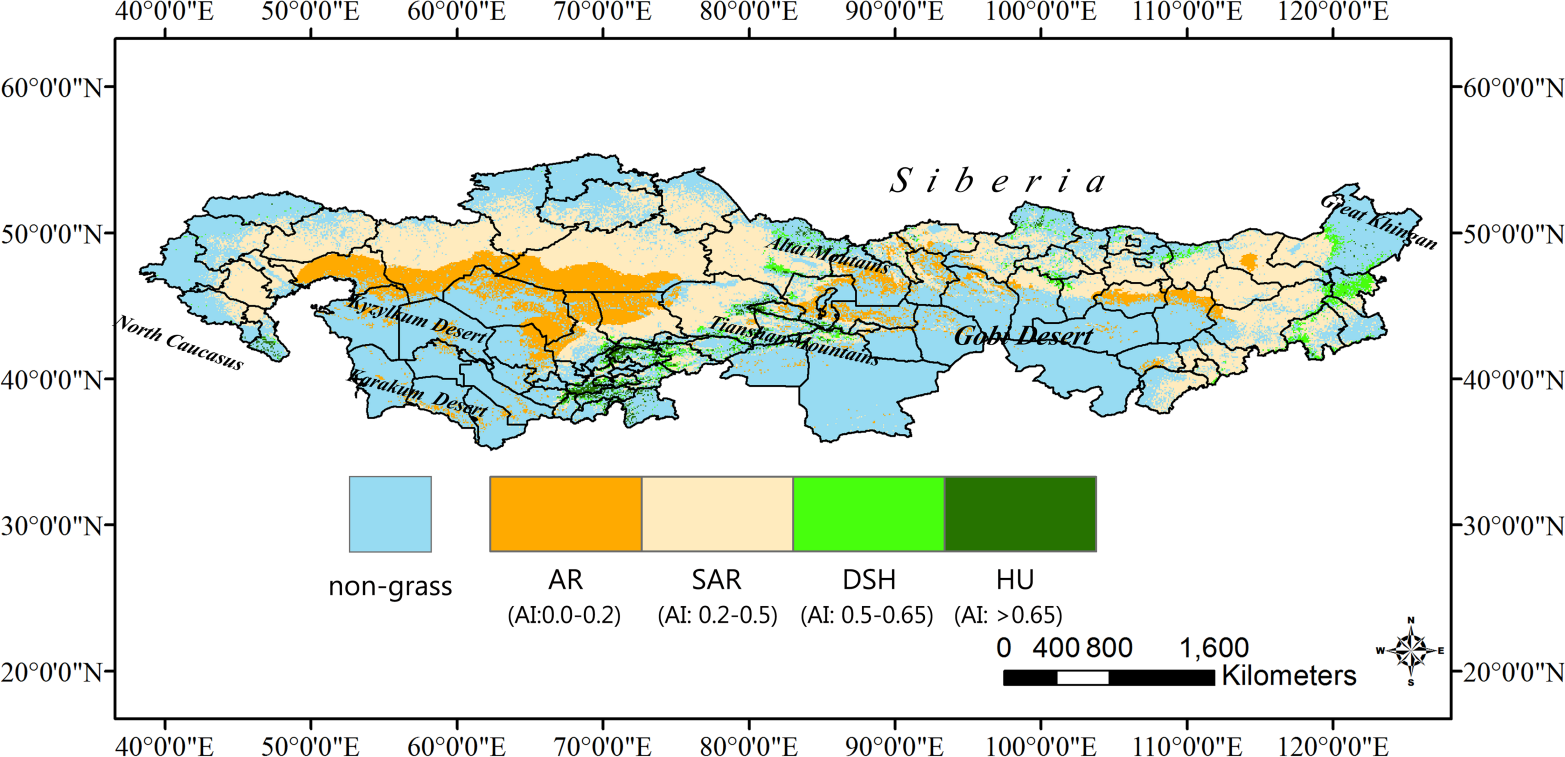
Map of study region classified by aridity index. AR: arid sub-region, SAR: semi-arid sub-region, DSH: dry and semi-humid sub-region, HU: humid sub-region.

Efforts have been done to understand the carbon and water cycles in this region at different spatiotemporal scales. Precipitation has been recognized as a primary limiting factor for vegetation biomass production in the arid and semi-arid ecosystems[17, 18], while the energy availability (i.e., radiation) becomes more important with the amelioration of water condition [19]. Further results indicate that the terrestrial water availability can significantly influence the ecosystem WUE. Site level study showed that ecosystem WUE stimulated with increasing precipitation in a temperate steppe of Inner Mongolia [20]. But the regional scale results of WUE exhibit a two-stage pattern in response to water availability [21–23]. That is, WUE first increases with lower drought stress but decreases under the humid environment. Meanwhile, studies also suggested that some vegetational indexes (e.g. LAI) could be important predictors of WUE [21, 24]. However, the terrestrial aridity condition has not been explicitly divided in the previous studies, which could induce certain concerns on the regional results. For example, in the widely studied “arid/semi-arid” of Inner Mongolia, the water conditions and grassland types are diverse, ranging from the humid meadow steppe in the northeast to an extremely arid desert steppe in the southwest. The aridity level varies greatly throughout the region **(Fig 1)**. If we simply use the entire region to study the effects of climatic change on dryland ecosystems, then the results could be affected by including those relative humid areas. Furthermore, a great imbalance of research exists in this region. Maestre, Salguero-Gomez [25] summarized that most regional studies about global change were conducted in the eastern and westernmost part of TES, including the Mongol Steppe and East-European Steppe, whereas other sub-regions in TES were largely under-investigated, especially the Kazakh Steppe. Therefore, it is necessary to investigate those less-researched areas to improve our understanding.

In order to better address the issues, we used a late version of Boreal Ecosystem Productivity Simulator (BEPS) to simulate the carbon and water cycles in TES. The prototype model is a multi-cycle combined terrestrial biogeochemical model and has been proved to work well in grassland research [26, 27]. To further improve the model performance in TES, we modified some important parameters and algorithms in NPP simulation. Thereafter, the long-term observation based Aridity Index (AI) was used to classify the region from arid to humid. In this way, the terrestrial water availability is considered into our regional study. By this work, we try to investigate the spatiotemporal trends of NPP, ET and WUE in TES in a period from 1999 to 2008, and explore the responses of carbon and hydrological cycles to climatic factors at different terrestrial aridity levels.

## Materials and methods

### Study region

The study region is located in the middle part of the Eurasian continent and extends from Volgograd Oblast, Russia in the west to Mongol Plateau in the east (35°45'N-54°28'N and 39°28'E-124°36'E) **(Fig 1)**. Due to the wide spatial extension, the physiographical, topographic, morphological, edaphic and climatic conditions are diverse. The region is typically dominated by temperate continental climate with different degree of aridity. For large part of the region, summer is hot with high average temperature from 20°C to 26°C, peak daily temperature can exceed 40°C, while in the coldest months, the temperature can reach -30°C. Precipitation is also very unstable, ranging around 150mm– 500mm per year from the near desert to near forest. With the diversity of environmental conditions, grass vegetation varies from meadow steppe (forest steppe) in the humid or semi-humid area, true or typical steppe in a semi-arid area, semi-desert steppe in arid climate, and desert grassland in hyper-arid area. In this study, we conducted in Transvolga-Kazakhstan Steppe (TKS) including steppes of Kazakhstan, Tajikistan, Uzbekistan, Kyrgyzstan, Turkmenistan and Volgograd Oblast, Russia, and Mongol Steppe (MS) including steppes of Mongolia, Inner Mongolia and Xinjiang, China.

### Model description

**Fig 2:**
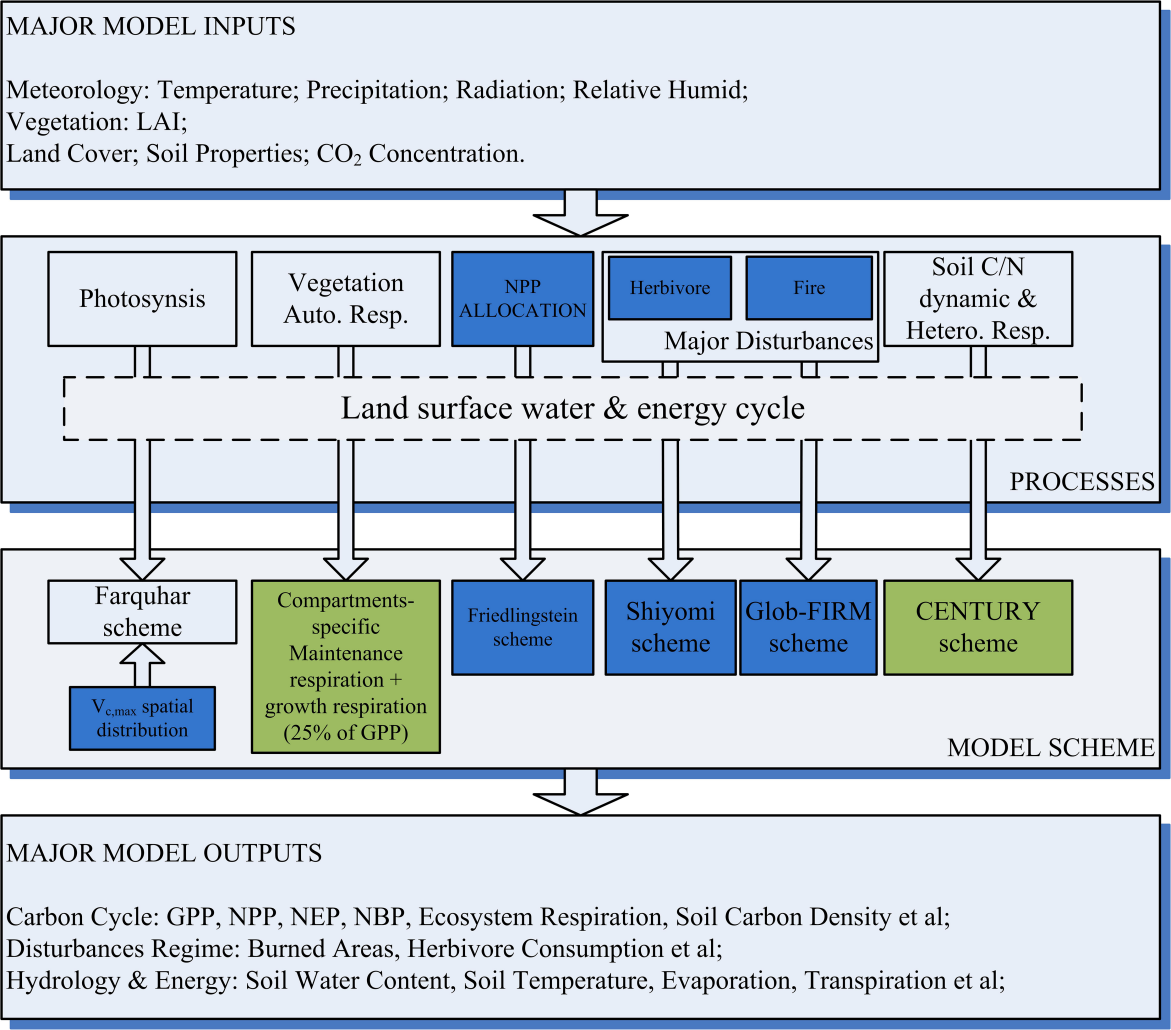
The schematic diagram of the revised BEPS. The blue boxes represent the model modifications and the orange boxes represent the major outputs analyzed in this study.

In this study, we modified and applied a late version of BEPS, which includes photosynthesis, soil biochemical and energy and hydrological balance modules (Fig 2). The model has been used widely to estimate regional or global terrestrial carbon and water fluxes [28–32]. Detail model descriptions could be found in previous studies [32–36]. Only NPP and ET calculations and major model modifications are introduced here.

#### NPP calculation

*The NPP* is calculated by subtracting vegetation autotrophic respiration (*R*_*a*_) from gross primary production (*GPP*):

$$NPP=GPP-R_{a}$$

The *GPP* is calculated with a “two-leaf” scheme. The canopy is separated into sunlit and sunshade. Sunlit and sunshade LAI can be achieved using total leaf area index (*LAI*) and clumping index (*Ω*) of specific vegetation type. The instantaneous photosynthesis rate is calculated by the Farquhar model [37]. The maximum carboxylation rate (*V*_*c*,*max*_) is an important parameter to determine the photosynthesis assimilation. Instead of using a preset value for the entire region, we incorporated a multi-factor based model from Zhang and Zhou [38]. The model was developed by compiling a series of experimental results from previous observations. The equations were established based on the statistical functions between observed *V*_*c*,*max*_ and each single environmental factors. The model has been successfully validated using multi-observation from various vegetation types [38, 39].

The model considers the effects of temperature, water, atmospheric CO_2_:

$$V_{c,\max}=V_{m,0}\times f\left(Temp\right)\times f\left(SW\right)\times f\left(CO_{2}\right)$$

Where *V*_*m*,*0*_ is the maximum carboxylation rate under an optimum environmental condition, *f*(*Temp)*, *f(SW)*, *f(CO*_*2*_*)* are functions of atmospheric temperature (Temp)(°C), surface soil water content(SW)(%), CO_2_ concentration(CO_2_) (Pa) that influence the value of *V*_*c*,*max*_.

$$f\left(Temp\right)=-1.67\times 10^{-3}Temp^{2}+0.11Temp-0.65$$

$$f\left(SW\right)=-4.4\times 10^{-4}SW^{2}+0.045SW-0.137$$

$$f\left(CO_{2}\right)=-1.08\times 10^{-4}CO_{2}^{2}+0.014CO_{2}+0.536$$

Temperature and CO_2_ concentration are from input data directly, the SW is from the model output.

*R*_*a*_ is separated into two parts: maintenance respiration (*R*_*m*_) and growth respiration (*R*_*g*_). *R*_*g*_ is estimated as proportions of GPP for different plant component according to previous studies. For *R*_*m*_, we replaced Bonan [40] equation in the original model with a component-specific scheme:

$$R_{m,i}=B_{i}\times p_{m,i}\times Q_{mr}^{\frac{\left(T-T_{r}\right)}{10}}$$

$$R_{g,i}=p_{g,i}\times A_{i}\times GPP$$

where *B*_*i*_ is a biomass of plant component *i*, *A*_*i*_ is a carbon allocation fraction in *i*, *p*_*m*,*i*_ and *p*_*g*,*i*_ are coefficients of maintenance respiration and growth respiration, respectively. *Q* is the temperature sensitivity factor of respiration calculated as function of temperature following Arora [41]:

$$Q_{mr}=3.22-0.046T$$

#### ET calculation

The ET is calculated following the ecosystem–atmosphere simulation scheme (EASS), by the combination of plant transpiration (*T*_*p*_), plant evaporation (*E*_*p*_), soil evaporation (*E*_*s*_), plant snow sublimation (*S*_*p*_) and soil snow sublimation (*S*_*s*_) based on energy conservation principle.

$$ET=T_{p}+E_{p}+E_{s}+S_{p}+S_{s}$$

As the “two-leaf” method in photosynthesis simulation, *T*_*p*_ is separated into sunlit and sunshade parts:

$$T_{p}=T_{p,lit}\times LAI_{p,lit}+T_{p,shade}\times LAI_{p,shade}$$

Where *T*_*p*,*lit*_ and *LAI*_*p*,*lit*_ are transpiration and LAI of sunlit leaves, and *T*_*p*,*shade*_ and *LAI*_*p*,*shade*_ are transpiration and LAI of sunshade leaves, respectively.

Then, T_*p*_ and E_*s*_ are calculated using the Penman-Monteith equation for no snow cover areas. For calculating *E*_*s*_, surface resistance is replaced by soil resistance according to Monteith [42].

*E*_*p*_ is calculated as following:

$$E_{p}=\min(S_{ci}\times A_{water}/LH_{v},P_{i})$$

Where *S*_*ci*_ is the intercept daily solar radiation (J m^-2^ day^-1^), *A*_*water*_ is the absorptivity of solar radiation for water, *LH*_*v*_ is the latent heat of vaporization (= 2.5×10^6^ J kg^-1^at 0°C) and *P*_*i*_ is the daily intercepted rainfall by canopy, which is determined as a proportion of LAI:

$$P_{i}=\min\left(LAI\times k_{i},PREP\right)$$

Where *k*_*i*_ is defined as the rainfall interception coefficient. The value is 0.3 mm LAI^-1^ d^-1^. *PREP* is the daily precipitation.

Sublimation only occurs when snow exists and *S*_*p*_ and *S*_*s*_ are estimated by following equations according to Liu [2003]:

$$S_{p}=\min\left(S_{ci}\times A_{snow}/LH_{s},P_{i}\right)$$

$$S_{s}=\min(SNOW,\left(S-S_{ci}\right)\times m_{snow}/LH_{s})$$

Where *A*_*snow*_ is the absorptivity of solar radiation for snow, *LH*_*v*_ is the latent heat of sublimation (= 2.8×10^6^ J kg^-1^ at 0°C), *SNOW* is the snow water equivalent (mm), *S* is the daily total income solar radiation and *m*_*snow*_ is a coefficient for the fraction of solar radiation transferred to latent heat by sublimation, which is set to 0.12 according to Saunders, Munro [43].

### Descriptions of sites for model validation

The NPP results were tested against the field measurement results. The observed data were collected from Eastern Kazakhstan (KS sites), Xinjiang (XJ sites) and Inner Mongolia (IM sites). Each of these sites represents a typical vegetation pattern in the local area. No specific permits were required for the described field studies because all field sites were located on public land. The biomass data were sampled from four replicates within test sites. Total biomass was defined as the sum of the herbaceous biomass, root, litter carbon pools. The peak-season living aboveground biomass and litter were measured by destructive sampling of 1.0 m^2^, roots were collected by excavating a square of 1.0 × 1.0 m to a depth of 50 cm. For each test site, productivity was calculated from five repeated observations at the sample plots. We both run the original model (i.e., BEPS) and the revised model to test if our modification could improve the model performance in the study area.

Four grassland eddy covariance (EC) sites’ data were used to validate the ET outputs. Three of them are located in Northern Kazakh Steppe and the other one is located in Inner Mongolia. Daily step data of latent energy (LE) with complete meteorological information were used to evaluate the corresponding model results. The data of three Kazakh Steppe sites were collected from the Fluxnet website (http://www.fluxdata.org/) and the data of Inner Mongolia site was offered by Meng Xiangxin and Fu Congbin. We selected the daily data of the EC sites with complete meteorological information needed for model simulation. All data were gap-filled and quality checked before the application.

The sites’ information is displayed in Table 1.

**Table 1:** List of site information for model validation.

Site	Data type	Long.	Lati.	Climate type^*^	Time extent	References
CN_TY	FLUX	122°52'E	44°25'N	Dwa	2008	Xiangxin and Congbin [44]
RU_HA1	FLUX	90°0'E	54°43'31"N	Dfc	2002–2004	Marchesini, Papale [45]
RU_HA2	FLUX	89°57'24"E	54°46'23"N	Dfc	2002–2003	Marchesini, Papale [45]
RU_HA3	FLUX	89°4'40"E	54°42'16"N	Dfc	2004	Marchesini, Papale [45]
KH (14 sites)	Field	72°43'56.0E-73°37'03.4"E	48°52'28.9"N-48°55'09.6"N	Dfb, Dfa,BSk	2004	Propastin, Kappas [46]
IM (54 sites)	Field	111°6'3"E-118°20'24"E	42°19'12"N -46°9'36"N	BSk,Dwb	2004–2008	Chen, Mu [47]
Xinjiang (52 sites)	Field	88°37'E-88°40'E	44°29'N-44°31’N	Bwk	2012	Chen, Mu [47]

* Climate type of grassland is based on Koeppen-Geiger classification (http://koeppen-geiger.vu-wien.ac.at/). BSk: main climate—arid, precipitation—steppe and temperature—cold arid; Bwk: main climate—arid, precipitation—desert and temperature—cold arid; Dfa: main climate—snow, precipitation—full humid and temperature—hot summer; Dfb: main climate—snow, precipitation—full humid and temperature—warm summer; Dfc: main climate—snow, precipitation—full humid and temperature—cool summer; Dwa: main climate—snow, precipitation—desert and temperature—hot arid; Dwb: main climate—snow, precipitation—desert, temperature—warm summer.

### Data preparation

#### Aridity index classification map

The AI map from the Consultative Group for International Agriculture Research (CGIAR) was used (http://www.csi.cgiar.org). The dataset follows the standard form proposed by United Nations Environment Programme (UNEP). It defines the AI as the ratio of Mean Annual Precipitation (MAP) to Mean Annual Potential Evapotranspiration (MAE):

AI=MAP/MAE

According to the original classification, terrestrial ecosystems are classified into 5 levels: hyper-arid (AI < 0.03, HAR), arid (0.03 < AI < 0.20, AR), semi-arid (0.20 < AI < 0.50, SAR), dry and sub-humid (0.50 < AI < 0.65, DSH) and humid (AI > 0.65, HU). However, few HAR areas exist in TES, so we combined that category with AR **(Fig 1)**.

#### Model input data

**Land cover map:** MODIS land cover product for 2001 (MOD12Q1, V004, IGBP global vegetation classification, 1km resolution, http://ladsweb.nascom.nasa.gov/data/) was used as model input because it is of high accuracy and has wide usage. The study region was extracted in Arc GIS 10.0. We used the version with the IGBP global vegetation classification. The TES map was extracted and resampled to an 8km resolution to fit the model input.

**LAI:** We used a daily step 8km LAI product (http://www.globalmapping.org/globalLAI/) to drive the model. The dataset derived from MODIS and AVHRR based on quantitative fusion algorithm. MODIS LAI was prepared from MODIS product (MOD09A1). Then a relationship between AVHRR SR and MODIS LAI was established using the overlapping period (2000–2006). Detail processes can be seen in Liu et al. [2012].

**Daily meteorological data:** Daily temperature, precipitation, radiation and specific humidity are required to drive the model. We imported the dataset of Global Meteorological Forcing Dataset for Land Surface Modeling (http://rda.ucar.edu/datasets/ds314.0/). The dataset is based on global observation datasets and NCEP/NCAR reanalysis. And it was updated and improved recently by using the results from the World Meteorological Organization (WMO) Solid Precipitation Measurement Inter-comparison and Global Precipitation Climatology Project (GPCP) daily product. Then it was evaluated by the second Global Soil Wetness Project (GSWP-2) dataset. The original dataset of precipitation and temperature were superimposed on re-sampled 8km Worldclim data generated from weather stations to reduce the deviation from spatial variation of topography. The regional MAT and MAP outputs in this study are also based on the daily temperature and precipitation from this dataset.

**Atmosphere CO**_**2**_ **data:** Monthly atmospheric CO_2_ data were collected from Mauna Loa Observatory (MLO), Hawaii (20°N, 156°W) (http://cdiac.esd.ornl.gov/ftp/trends/co2/maunaloa.co2). The atmospheric CO_2_ concentration is assumed to be uniform across the region.

**Soil texture data:** Soil texture data were collected from the Global Soil Dataset for use in Earth System Models (GSDE, Available at: http://globalchange.bnu.edu.cn/research/soilw). The data are displayed as the volumetric percentages of silt, clay and sand. The original spatial resolution is 30 arc-seconds (about 1 km at the equator) and was bi-linearly interpolated into 8 km resolution.

The protocol of this study is available at: dx.doi.org/10.17504/protocols.io.h3mb8k6.

## Results

### Spatiotemporal distribution of meteorological conditions in TES

**Fig 3:**
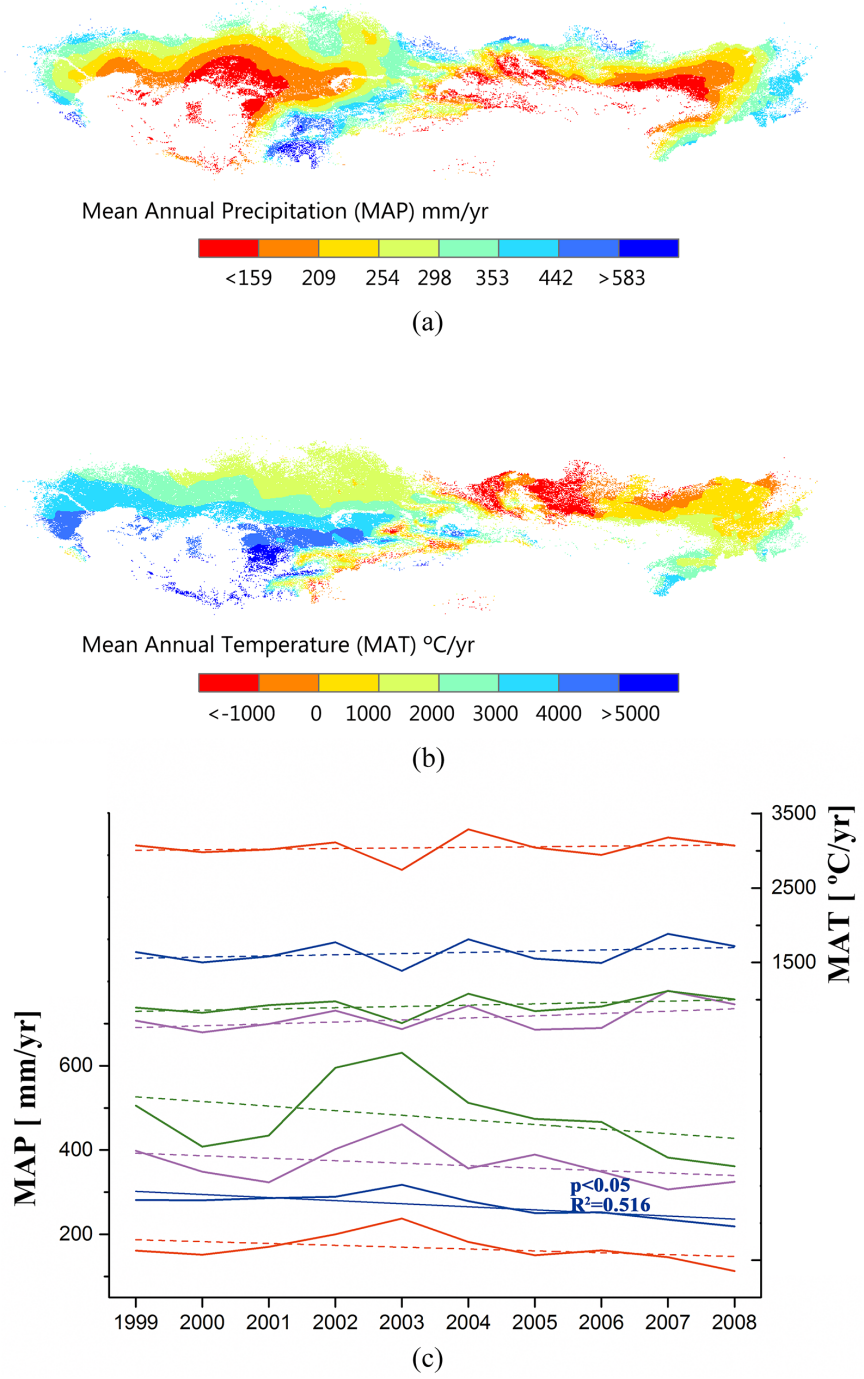
Regional meteorological conditions and variations from 1999 to 2008 in Temperate Eurasian Steppe. (a) Spatial distribution of mean annual precipitation (MAP); (b) Spatial distribution of mean annual temperature (MAT); (c) temporal variations of MAP and MAT from 1999 to 2008 in different AI sub-regions: AR (red lines), SAR (blue lines), DSH (magenta lines) and HU (green lines).

Mean annual temperature (MAT) shows an obvious zonal characteristic from AR to HU sub-regions. **(Fig 3A)**. High temperature areas are in the desert neighboring areas such as the Kyzylkum Desert, the Karakum Desert and the Gobi Desert, which are mainly characterized as AR. Low temperature areas are in the northwestern part of Mongolia, the northeastern part of Kazakhstan, the mountainous Altai region connected to Siberia in Russia, and the Tianshan Mountains, which stretches over Kyrgyzstan, Tajikistan and China. During the study period, the MAT of all sub-regions was increasing but no significant trend was found **(Fig 3C)**.

Mean annual precipitation (MAP) increases from AR to HU, ranging from 167.3mm to 477.1mm **(Fig 3B and 3C)**. Deserts bordering areas of AR experience little precipitation (<100mm). High MAP areas consist of HU, DSH and scattered SAR near the northwestern border with Russia, Mongolia, Inner Mongolia in China, and the open mountain grasslands and alpine meadows in Central Asia. The MAP in HU and DSH showed greater variations than those in relatively barren areas. Decreasing MAP trends were found in all sub-regions. However, only the MAP in SAR decreased significantly during the decade (p < 0.05, R^2^ = 0.516).

### Validation of simulated results

**Fig 4:**
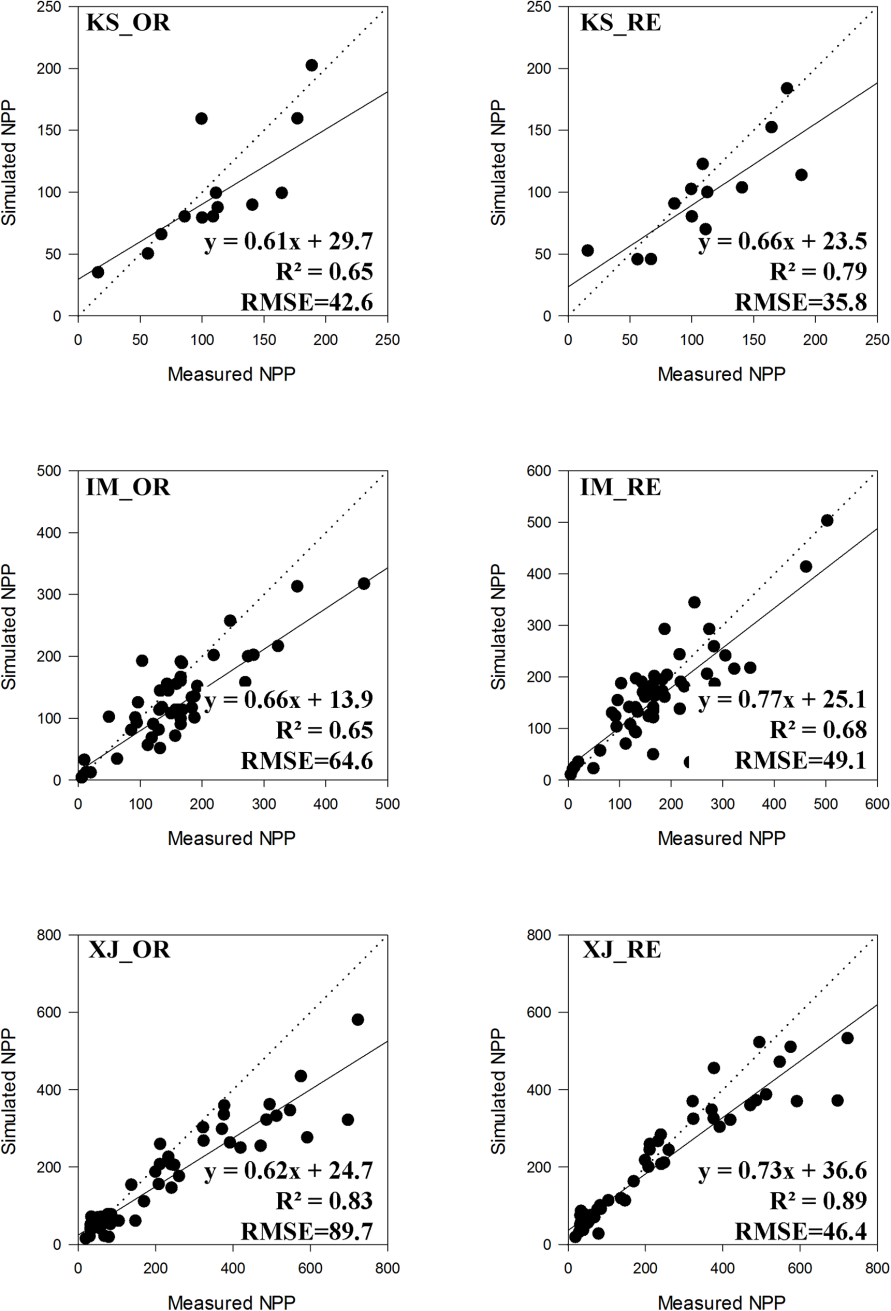
Comparisons between simulated NPP and measured NPP from independent field study in Kazakh Steppe (KS), Inner Mongolia (IM) and Xingjiang (XJ): (a) the result of the original model (OR); (b) the result with revised model (RE).

Comparisons between modeled NPP and observed NPP for the three sets of field measurements are showed in **Fig 4**. According to the results, the NPP from the revised model exhibited good agreements with field measurements in all three groups of sites. The R^2^ were 0.79, 0.68, 0.89 for the sites in Kazakh Steppe (KS), Inner Mongolia (IM) and Xinjiang (XJ), respectively. The RMSE were 35.8 gC/m^2^, 49.1 gC/m^2^ and 46.4 gC/m^2^ for the KS, IM and XJ sites, respectively. For the model inter-comparison, the results of revised model performed better than the original model in all three sites. The correlation in KS showed the highest R^2^ increase of 0.14 (0.79 versus 0.65), the corresponding R^2^ increases were 0.06 and 0.03 for the XJ and IM sites, respectively. The RMSE decreased by 6.8 gC/m^2^, 15.5 gC/m^2^ and 33.3 gC/m^2^ for the KS, IM and XJ sites, respectively.

**Fig 5:**
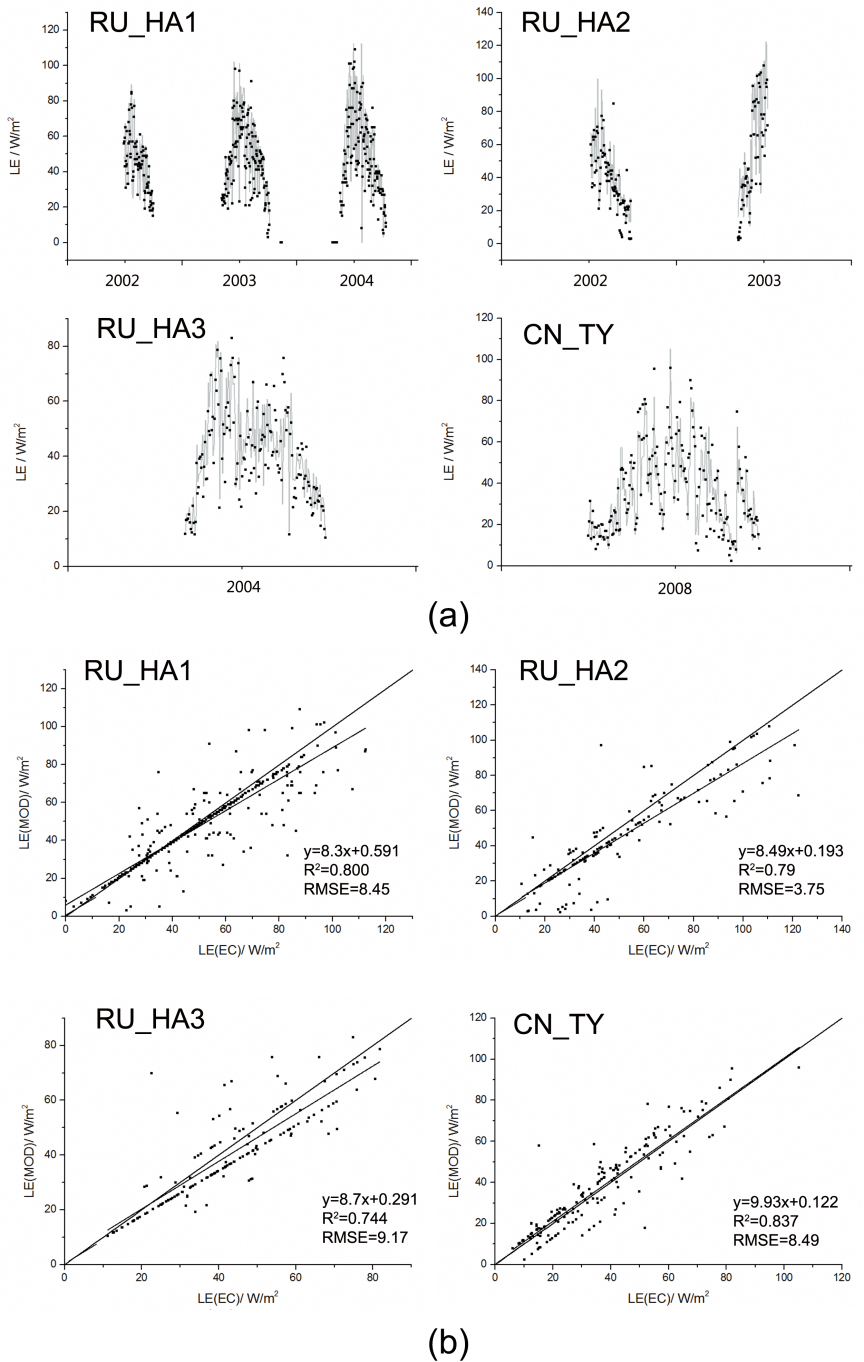
Validation of modeled LE (LE_MOD_) using the daily eddy covariance data (LE_EC_) at 4 sites (RU_HA1, RU_HA2, RU_HA3, CN_TY).

**Fig 5** shows the LE comparisons between the observations and simulations in the EC sites. The average R^2^ was 0.79, ranging from 0.744 (RU_HA3) to 0.837 (CN_TY). The average RMSE was 7.47 W/m^2^, ranging from 3.75 (RU_HA1) to 9.17 W/m^2^ (RU_HA2).

The above statistical comparisons indicate that our modifications could improve the model performances and the model results are credible and suitable for this regional study.

### Spatiotemporal distributions and variations of NPP, ET and WUE

**Fig 6:**
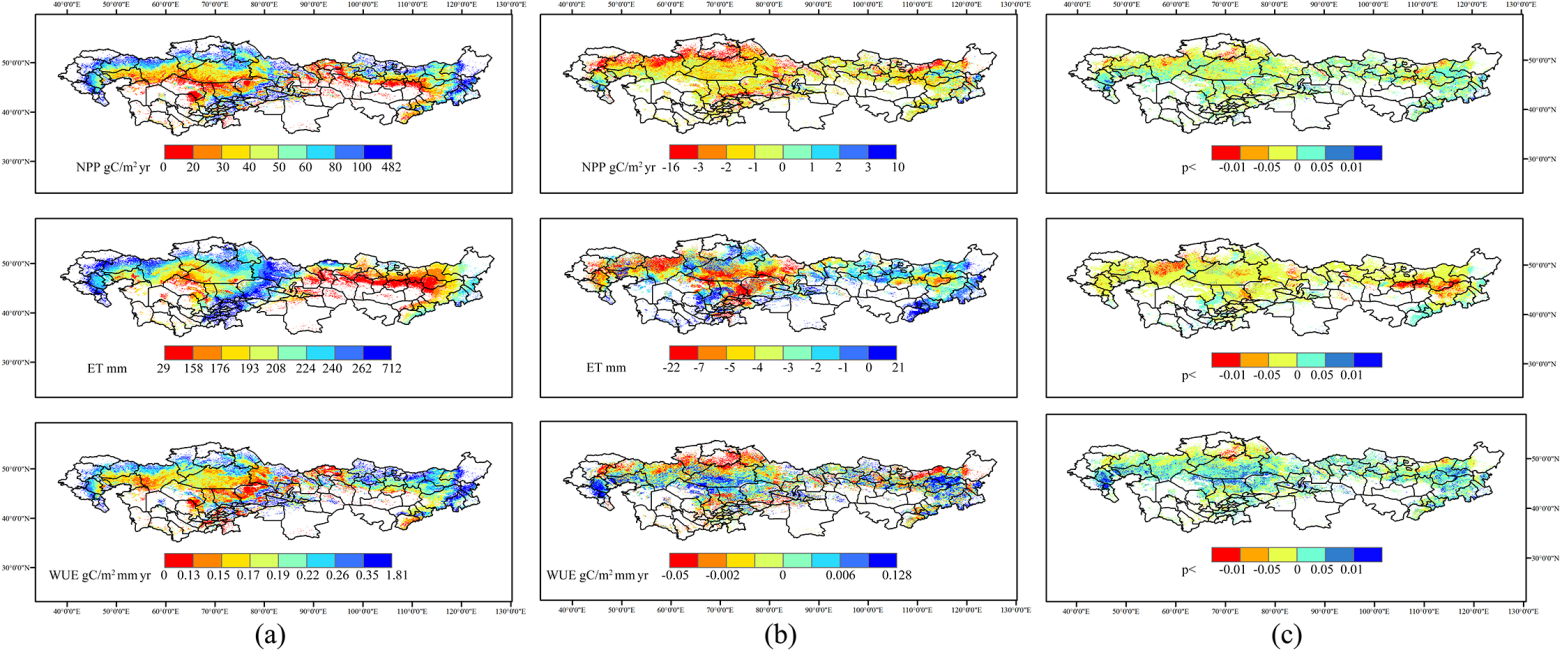
Regional spatial distributions of (a) mean annual value, (b) annual change value and (c) temporal significance of NPP, ET and WUE in Temperate Eurasian Steppe from 1999 to 2008.

**Fig 7:**
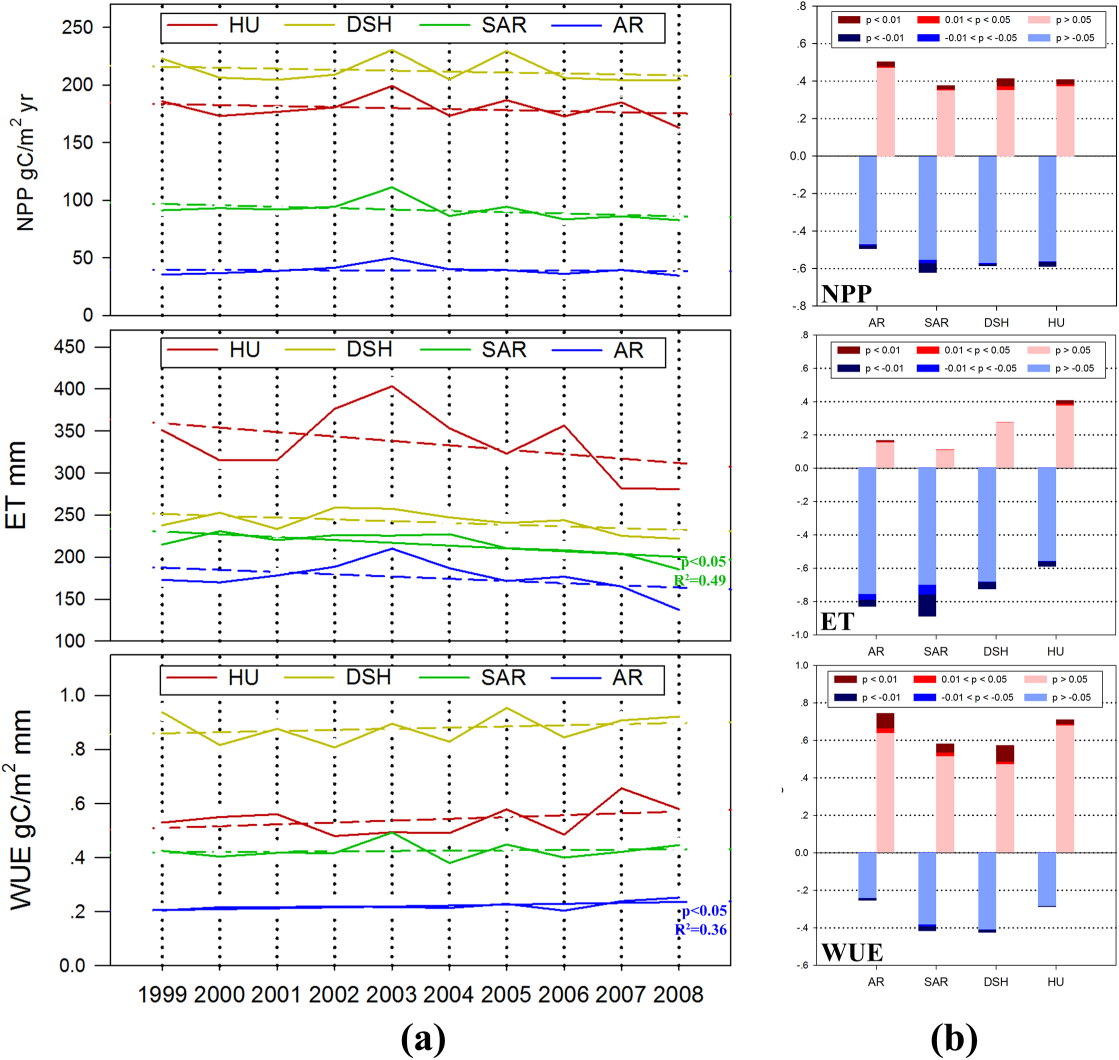
Temporal trends of NPP, ET and WUE from 1999 to 2008 in the Temperate Eurasian Steppe under different AI classification: (a) trend of the regional annual mean; (b) area percentage with an increase or decrease trend.

The spatiotemporal distributions and variations of the NPP, ET and WUE are showed in **Fig 6** and **Fig 7**. There was no significant temporal trend of the NPP in each sub-region during the 1999–2008 period **(Fig 6A)**. However, the mean values were very different across the four sub-regions. The annual NPP of DSH and HU were significantly larger than AR and SAR. The highest value of 212.3 gC/m^2^ was in DSH and the lowest value of 39.16 gC/m^2^ was in AR. The NPP shows a clear zonal distribution from near-forest to near-desert **(Fig 6A)**. High productivity grassland mainly concentrated in these areas connected with the forested areas, such as meadow steppe in the east part of Mongol Steppe, which is joined to Great Khingan and Siberia, alpine-meadow steppe around Tianshan and Altai Mountains, and Russian steppe areas that are close to the North Caucasian Forest. The NPP of these areas are generally higher than 100 gC/m^2^ per year. During the decade, The NPP reductions were found in many parts of these areas, especially the Northern part of Kazakhstan and the alpine meadows near the mountainous area in Central Asia. The NPP mainly increases in the grassland of Inner Mongolia and Russia. Grasslands with lower productivity are those areas near the deserts (mainly with AR dominated), for which the NPP are typically lower than 30 gC/m^2^ per year. In these barren areas, annual NPP variations were smaller and the trends were less identifiable **(Fig 6B and 6C)**. According to the regional statistics, during the period, more than 60% of the total areas showed a decreasing trend of NPP **(Fig 7B)**.

The largest mean annual ET was 335.7 mm in HU, which was significantly higher than the other sub-regions. The values in DSH, SAR and AR were 241.9mm, 215.28 mm, 175.65 mm, respectively. The ET showed decreasing trends in all four sub-regions (i.e., arid, semi-arid, dry and semi-humid and humid sub-regions), but the trend was significant (P<0.05) only in SAR **(Fig 7A)**. Grasslands with high ET are located in the mountainous Tajikistan, Kyrgyzstan, Eastern and western part of Kazakhstan **(Fig 6A)**; Grasslands with moderate ET are located in the areas with high precipitation but with relatively low temperatures, such as the eastern part of Inner Mongolia. The ET decreased in more than 80% areas of TES during the decade **(Fig 7B)**. The largest reduction was found in SAR, approximately 10% areas of the sub-region showed significant decreasing trends. They were mainly distributed in the Southwestern part of Mongolia. The largest annual reduction was found in the Central Kazakh Steppe **(Fig 6B and 6C)**.

During the decade, the largest mean annual WUE was found in DSH, with a value of 0.88 gC mm^-1^, and the lowest value was found in AR with 0.22 gC mm^-1^. The inter-annual trends of WUE were not significant in all sub-regions except AR, where the WUE showed a significant enhancement (p < 0.05) **(Fig 7A)**. An overall increasing trend of WUE was found throughout the region. Exceptions were some areas near the northern borders in Kazakhstan and Mongolia, where the NPP decreased significantly. However, even for those areas with negative trends, their WUE reductions were very small **(Fig 6C)**. The largest WUE increases were found in AR, where nearly 80% areas showed increase trends and more than 10% of the areas exhibited significant (p < 0.05) or very significant trends (p < 0.01) **(Fig 7B)**.

### The response of NPP, ET and WUE to climatic factors

**Fig 8:**
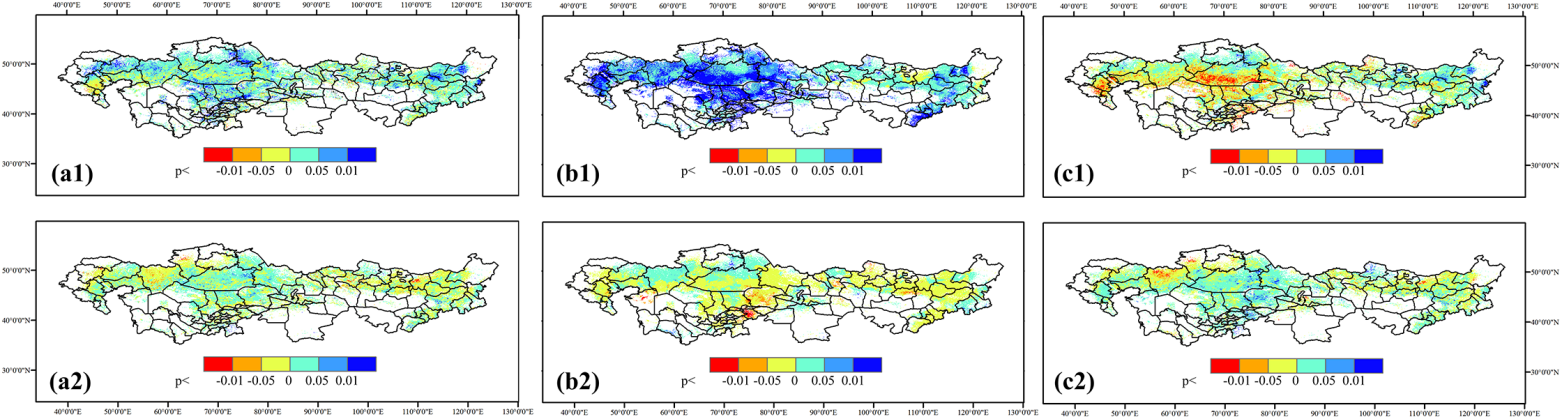
Spatial distribution of correlationships between (a1) NPP and MAP, (a2) NPP and MAT, (b1) ET and MAP, (b2) ET and MAT, (c1) WUE and MAP and (c2) WUE and MAT in the Temperate Eurasian Steppe under different AI classification.

**Fig 9:**
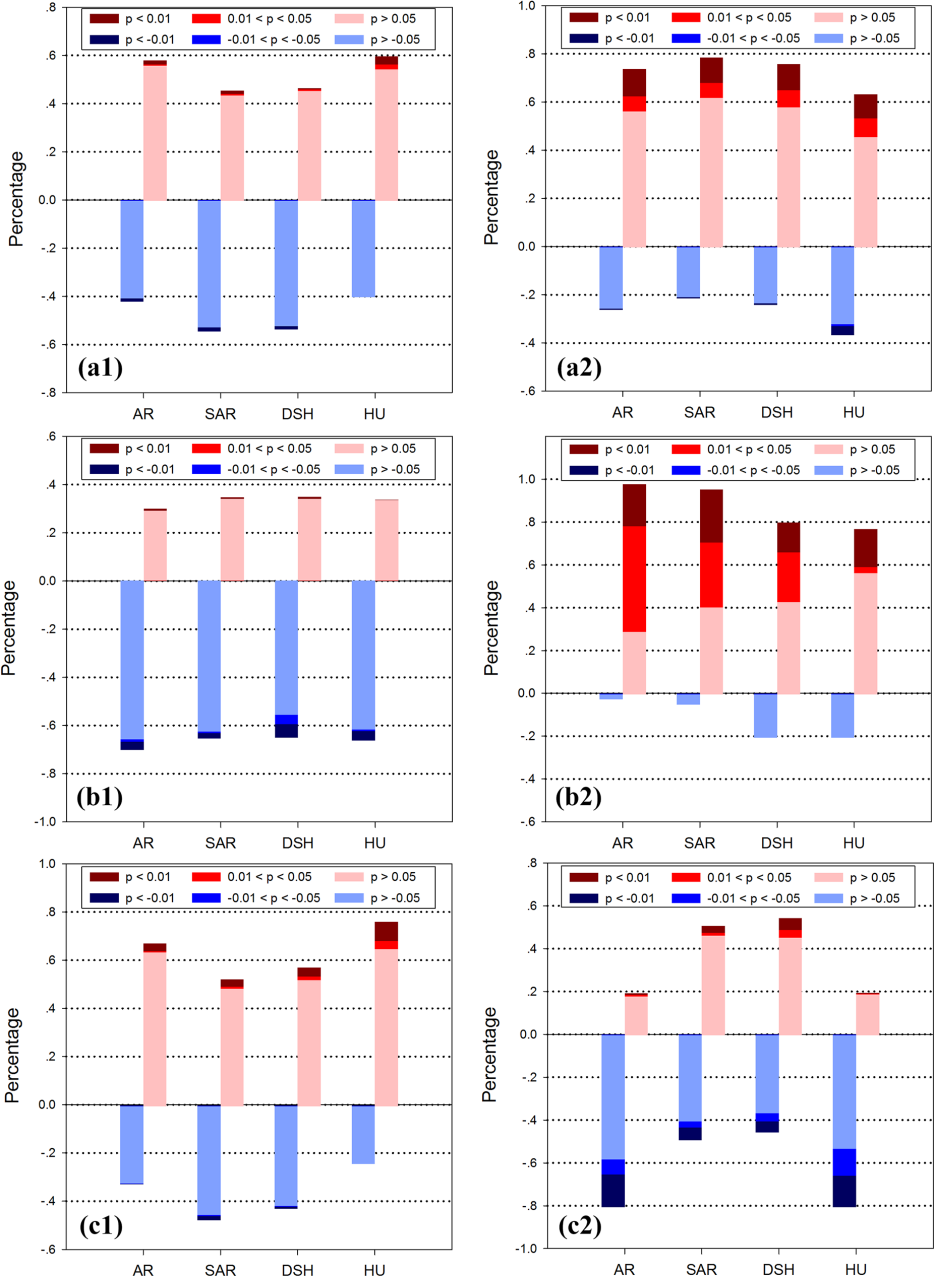
Area percentage of the correlationship between (a1) NPP and MAT, (a2) NPP and MAP, (b1) ET and MAT, (b2) ET and MAP, (c1) WUE and MAT and (c2) WUE and MAP in the Temperate Eurasian Steppe under different AI classification.

Spatial distribution of correlationships between the NPP, ET and WUE and the climatic factors are displayed in **Fig 8**. The results showed that the correlationships between the MAT and NPP were ambiguous throughout the region. The positive relationships were mainly found in HU. The ET were negatively correlated with the MAT, which could come from the negative relationship between temperature and precipitation [48]. Significant correlations were found in the mountainous southern part of Kazakh Steppe. For the WUE, the grasslands in HU showed higher positive correlationships with the MAT than the other sub-regions **(Fig 9)**.

The NPP were positively correlated with the MAP in all four sub-regions. The areas with significant correlationships were mainly in the semi-arid Central part of Mongolia, the Northern part of and the Western part of Kazakhstan and the desert steppe in the Central part of Kazakhstan **(Fig 8)**. The regional ET exhibited an overall significant positive correlationship with the MAP, especially in the Kazakh Steppe. The significance of the correlationships clearly increased with aridity level. The area percentage of significant positive correlationship ranged from 68.7% in AR to 20.3% in HU **(Fig 9)**. For the WUE, more than 80% areas in AR and HU were negatively correlated with the MAP, which were mainly in the Central and South end of Kazakh Steppe. The correlationships were not very clear in SAR and DSH sub-regions.

## Discussion

### Evaluation and comparison of the model results

In this study, our modified BEPS was tested and used to simulate the carbon and hydrological cycles in TES. The model was validated against multi-source datasets, which indicated its ability to accurately simulate the regional grassland ecosystem. Research comparison shows that our NPP results are more comparable with those models that share the same canopy photosynthesis scheme (i.e., Farquhar biochemical model), but much lower than the result from the CASA model (a light use efficiency model). However, it seems that the CASA model over-estimates NPP because the value declined significantly after an improvement by Xing, Xu [49]**(Table 2)**. Furthermore, different uses of input data could be another source of difference. For example, for BEPS, if topography is considered to interpolate meteorological data, the production decreases significantly in grassland simulation [50]. The soil effects in sparse vegetation region cause the bias of the MODIS LAI model in arid/semi-arid areas [51, 52].

**Table 2:** Result comparison between recent studies and modeled results of this paper for net primary productivity (NPP) and evapotranspiration (ET). NPP unit is gC m^-2^ yr^-1^ and ET unit is mm.

Study area	Study period	Method	Results	Corresponding results in this study^*^	reference
Mongolia	2000–2005	Improved CASA model	NPP: 125.33	75.2	Xing, Xu [49]
Northern China	2000–2005	Improved CASA model	NPP:153.26	113.8	
China	2000	CASA	NPP:245	121.23 (for Inner Mongolia)	Gao and Liu [53]
		CEVSA	NPP:208		
		GLOPEM	NPP:145		
		GEOLUE	NPP:178		
		GEOPRO	NPP:168		
China	2007	BEPS	NPP: 122.6	121.23 (for Inner Mongolia)	Feng, Liu [50]
Eastern Kazakhstan	2004	Modified light use efficiency model	NPP:168	152.1	Propastin and Kappas [54]
East Asia	1982–2006	BEPS	ET:243	209.84 (for TES from 1999 to 2006)	Zhang, Ju [21]
Xinjiang and Central Asia	2005	SEBS	ET:174.85	178.82	ABDULLA [55]
Inner Mongolia	1982–2009	Revised RS-PM model	ET: around 210	197.85	Li, Verburg [56]
Xingjiang	1982–2009	Revised RS-PM model	ET: around 190	179.8	
Xilin river catchment in Inner Mongolia	2006	BROOK90	ET: 202	190.7	Schaffrath, Vetter [57]

*the results are extracted from the simulated map in this study with the same spatiotemporal scale to the corresponding recent studies. The results are mean annual values of the corresponding study period.

Regarding to the ET results, the results produced in this study are very similar to the results from other remote sensing based models.

### WUE at different AIs and their responses to climatic change

Different WUE were found under different AIs. The WUE in TES showed in a decreasing order of: DSH (0.88 gC m^-2^ mm^-1^) > HU (0.54 gC m^-2^ mm^-1^) > SAR (0.43 gC m^-2^ mm^-1^) > AR (0.22 gC m^-2^ mm^-1^). The result indicates that WUE could increase with lower aridity stress in a certain range, but decline under the humid environment. This two stage pattern corresponds to the regional results in the conterminous US and East Asia [21, 58].

According to the AI classification, TES mainly consists of SAR (60.48%) and AR (23.14%). The AR and SAR sub-region are typically with high MAT and low MAP according to our regional investigation (**Fig 3**). The continentality is more obvious in the Kazakh Steppe due to its longer distance from the sea. In this large water-deficient region, the climate continues to become drier. The temporal results indicated that during the decade, the temperature did not show statistically significant trend, but the warming tendency is obvious over all the sub-regions. At the longer temporal scales, the regional warming has been agreed by all the observations available. The steady significant warming trend has been recorded for more than 60 years in Central Asia [59]. According to the latest IPCC report [1], Northern Eurasia is among the land areas with the strongest warming signals. Chen, Wang [60] further indicated that the temperature increase magnitude in this region is approximately twice as the average of the entire Northern Hemisphere. Comparing to the general consensus on the regional warming, the precipitation pattern is more variable. In the study decade, the temporal trend is varied over the different sub-regions. While in the dominated sub-regions (i.e., SAR and AR), MAP tends to decrease more significantly. The decadal pattern consists with the centennial regional analysis from Ma and Fu [61] and a global investigation of drylands [25, 62]. Our results also support the predictions from Rind, Goldberg [63], who indicated that the precipitation in mid- and high- latitude of North Hemisphere would decline due to the projected climate change. However, Chen, Huang [64] reported that MAP was slightly increasing during 1930–2009 in the Central Asia. Nevertheless, all these studies agreed that the variability of MAP in arid/semi-arid areas is and will be increasing. The increasing variability of MAP, coupled with the regional temperature rising triggered the warm and dry tendency.

Under this condition, the regional WUE exhibits an increasing trend. However, it could not be concluded directly that the grasslands were getting better. Based on the regional results, we even find the decreasing trends of NPP in many areas. Hence, the regional increasing of WUE was mainly attributed to the ET reduction based on our results (Fig 6). This trend is more significant in the arid areas, especially the AR and SAR areas in the Northern part of Kazakhstan and the Southern part of Mongolia. Considering the good positive correlationship between MAP and ET, the decreased regional MAP could, therefore, indirectly induce the WUE enhancement. Besides, the ecosystem resilience to drier environmental condition could also contribute to the WUE increase [65]. For those species adapting to the extreme environment, they have developed strong stress tolerance abilities to survive effectively, *e*.*g*., reducing autotrophic respiration, conserving production into the soil and decreasing transpiration by stomatal regulation in response to the climatic deterioration [66–68]. Ecosystem evidence is that the NPP in AR showed higher resilience than the other sub-regions. In fact, only some of these resistance mechanisms were captured by the model via the LAI input and description of physiological and biochemical processes (*e*.*g*. NPP allocation, vegetation autotrophic respiration, and VPD calculation). Some other proved mechanisms are beyond current model prediction. The special aboveground morphology (e.g., leaf degradation) could enhance carbon assimilation efficiency with small LAI; the root structure of desert plants facilitates water absorption ability from deeper soils [69–71]. These processes secure the successful survive of desert plants under harsh environment conditions. Zhang, Li [70] further suggested that over-simplification of these processes in the terrestrial models would cause certain under-estimation of carbon sequestration in Central Asia. Furthermore, the prolonged drier environmental condition could also lead to the shift of species composition to become greater resistant to the drought events [72]. The physiological properties of drought-tolerant species could generate higher rates of water and carbon dioxide exchange, which is benefit for ecosystem functioning and sustainability [73, 74]. Hence, the resilience effect of the arid ecosystems should be stronger than our current prediction.

Currently, the CO_2_ fertilization effect is regarded as an important contributor to the increasing C accumulation in terrestrial ecosystems[75, 76]. Its impact to the temperate grasslands, however, is not very clear. Based on the global satellite observations, Donohue, Roderick [77] showed that 14% increase in atmospheric CO_2_ led to a 5 to 10% increase in C assimilation in warm, arid environments. While the regional studies indicated that CO_2_ fertilization might only play a minor role in the temperate grassland ecosystems. Mu, Zhao [78] indicated that although CO_2_ fertilization has a strong impact on the carbon assimilation, its impact on temperate grasslands is the weakest with a contribution of only 0.3% of the total NPP variations. The results from the TEM showed that CO_2_ fertilization could only counteract 1.4% of a NEP decrease [79]. The study also suggested that the greatest part of C assimilation increasing attributed to a rise in CO_2_ was distributed in the sub-tropical and tropical ecosystems. Considering that CO_2_ fertilization has little impact on ET [80], we suggested that the CO_2_ fertilization will lead to the increasing of WUE, but the extent of this impact varied spatially. Further study is largely needed to promote our understanding to this process.

The temporal period in this study avoids the significant political impacts from the huge land revolutions like the collapse of the former USSR. However, the impacts of land use and cover change (LUCC) on carbon and water cycling still widely exist over the region. The current LUCC and its spatial heterogeneity are closely related to the human activity and governmental decisions. On the one hand, inappropriate human use and activities induce grassland degradation in arid/semi-arid areas. Human dominated activities like animal husbandry are regarded as the major factors leading to the negative land conversions [25, 81]. In some traditional fine pastures (e.g. Xilinhot Steppe and Hulun Buir Steppe), the quality of grass resource is much worse than the nomadic period [82, 83]. According to the assessment from Yusupov[84], overgrazing is the major factor to account for the land degradation in Central Asia with a contribution of 44%. Because of the recent boom of economic development, the impacts from other human activities such as mining and oil production are increasing as well[85, 86]. On the other hand, the regional conservation programs were settled to address the land degradation problems. The most well-known projects are from the Chinese government. induced a land recovery from deserts to grasslands or forests [87]. Recent evaluation pointed out that the grassland area increased by 77,993 km^2^ from 2000 to 2009, with 29,432.71 Gg C. yr^−1^ carbon accumulation increase in Inner Mongolia [88]. In addition, the current trend of urbanization moves a large amount of the rural population to the cities and contributing to the recovery of grasslands in the rural areas [89, 90]. These factors thus interact with each other and influence the regional pattern of WUE. Further assessments of the LUCC impact will be largely needed in the future studies.

The role of water to grassland ecosystems varies according to different regional features of vegetation physiology and adaptation. The plant growth in arid ecosystem is mainly limited by the vegetational limitations, such as the high root to shoot rate and the low stomatal conductance. These biophysical features are unavoidable trade-offs between the survival in the extreme environment and the higher growth rate. This type of limitation weakens with a more favorable water condition, while the biochemical limitations such as light and nutrient inputs become stronger. Interestingly, we found that in this region, the WUE shares a similar pattern of the MAP correlationship in AR and HU sub-regions. We consider that this similarity should be attributed to different underling mechanisms. For the humid areas, precipitation is sufficient for vegetation growth and transpiration, so too much water input can induce ineffective water use and higher biogeochemical requirements. For example, Austin and Vitousek [91] showed that foliar nutrient availability decreased with increasing annual precipitation, which indicates the shift of major limitation to biochemical constrains. Thus, we found that in HU, the NPP showed the weakest correlation with the MAP. For the areas in AR, which is mainly characterized with desert steppe, both the vegetation production and evapotranspiration are limited by vegetational constraints (e.g. LAI). Hence both of them increase with extra water inputs. Our results corroborate the conclusions from [92–94], who proposed that in the arid and semi-arid lands, NPP is closely correlated with precipitation and hence the rain-use efficiency is a good indicator of NPP. With great potential to lose water in AR, the ET increases almost linearly in a wide range of water input [41]. However, vegetation production cannot increase continuously to match ET due to the intrinsic limitation from vegetation physiology and increasing nutrient deficiency [95, 96]. Therefore, the combination induced the negative correlationship between MAP and WUE. Likewise, the response pattern is similar in SAR sub-region. However, for this sub-region, the more favorable environment allows higher vegetation growth potential, and the water loss potential is weaker than that of AR sub-region **(Fig 9)**. As a result, the corresponding response of WUE to MAP is less significant.

## Conclusion and implications

In this study, we modified and used BEPS to simulate carbon and hydrological cycles in a typical dryland ecosystem, Temperate Eurasian Steppe. The Aridity Index classification contributes to specifically distinguish areas under various terrestrial water availability conditions and reveal different responses and underling mechanisms to climatic change. Results showed that this region tends to become drier with the increasing temperature and decreasing precipitation. Although regional WUE showed an increasing trend, it could not be concluded that grassland was recovering since regional NPP was decreasing. We consider that the trend comes from a combination of ET reduction induced by lower precipitation, and ecosystem resilience to drier environmental condition. According to the prediction from the multi-model ensemble-mean [97], the regional aridity, persistent droughts and extreme climate will increase. In the long run, climatic change will lead to further vegetation degradation and deteriorative land cover conversions, e.g. desertification. The aridity aggrandizement will, thus, put more influence on multi-aspect of grassland ecosystem, *e*.*g*. carbon sequestration, soil quality and species biodiversity. Since TES mostly covers the developing countries, both the water resources and the grassland ecosystem are essential to the human-being living and social wellness [98, 99]. We have already noticed the importance to protect the grassland, but how to manage and preserve its ecological services and functions efficiently should be the next task for both international scientific community and local governments.

We find the two-stage pattern of WUE and the various responses to mean annual precipitation under different aridity levels in this region. The different responses in AR and SAR should also be noticed in future studies. We believe our findings could contribute to the mechanical understanding of carbon and hydrological circulations in dryland ecosystems and offer evidences to future terrestrial model development and improvement.

## Supporting information

S1 File The websites to achieve data used in this study. (DOCX)
