# Supplementary material for: Quantitative assessments of water-use efficiency in Temperate Eurasian Steppe along an aridity gradient
Source: PLoS One. 2017 Jul 7;12(7):e0179875. doi: 10.1371/journal.pone.0179875 (PMC5501447; doi:10.1371/journal.pone.0179875)
Supplement: S1 File — (DOCX) [file pone.0179875.s001.docx]

**The websites to achieve data used in this study**

**The data used in this study is available at the following websites:**

The Aridity Index classification map: <http://www.csi.cgiar.org>

MODIS land cover product for 2001: <http://ladsweb.nascom.nasa.gov/data/>

GlobalLAI dataset: <http://www.globalmapping.org/globalLAI/>

Global Meteorological Forcing Dataset for Land Surface Modeling: <http://rda.ucar.edu/datasets/ds314.0/>

Monthly atmospheric CO2 data from Mauna Loa Observatory (MLO): <http://cdiac.esd.ornl.gov/ftp/trends/co2/maunaloa.co2>

Global Soil Dataset for use in Earth System Models: <http://globalchange.bnu.edu.cn/research/soilw>

Eddy covariance (EC) sites’ data: http://www.fluxdata.org/
